# Clinical characteristics of the first known cases of death caused by COVID-19 pneumonia

**DOI:** 10.18632/aging.104171

**Published:** 2020-11-20

**Authors:** Fan-Zhen Kong, Yi Wang, Mei-Xia Wang, Qing-Zhang Cheng, Robert Logan, Guan-Hui Wu, Si-Ming Hu

**Affiliations:** 1 Department of Psychological Nursing, Suzhou Guangji Hospital, The Affiliated Guangji Hospital of Soochow University, Suzhou, China; 2 Department of Pulmonary Medicine, Fifth People’s Hospital of Suzhou, The Affiliated Infectious Hospital of Soochow University, Suzhou, China; 3 Department of Neurology, Suzhou Municipal Hospital, The Affiliated Suzhou Hospital of Nanjing Medical University, Suzhou, China; 4 Department of Biology, Eastern Nazarene College, Quincy, MA 02170, USA; 5 Department of Respiratory and Critical Care Medicine, Suzhou Municipal Hospital, The Affiliated Suzhou Hospital of Nanjing Medical University, Suzhou, China

**Keywords:** clinical characteristics, death cases, COVID-19, pneumonia

## Abstract

Severe pneumonia caused by COVID-19 has resulted in many deaths worldwide. Here, we analyzed the clinical characteristics of the first 17 reported cases of death due to COVID-19 pneumonia in Wuhan, China. Demographics, initial symptoms, complications, chest computerized tomography (CT) images, treatments, and prognoses were collected and analyzed from the National Health Committee of China data. The first 17 reported deaths from COVID-19 were predominately in older men; 82.35% of patients were older than 65 years, and 76.47% were males. The most common initial symptoms were fever or fatigue (14 cases, 82.35%), respiratory symptoms, such as cough (12 cases, 70.59%), and neurological symptoms, such as headache (3 cases, 17.65%). The most common finding of chest CT was viral pneumonia (5 cases, 29.41%). Anti-infectives (11 cases, 64.71%) and mechanical ventilation (9 cases, 52.94%) were commonly used for treatment. Most of the patients (16 cases, 94.12%) died of acute respiratory distress syndrome (ARDS). Our findings show that advanced age and male gender are effective predictors of COVID-19 mortality, and suggest that early interventions to reduce the incidence of ARDS may improve prognosis of COVID-19 pneumonia patients.

## INTRODUCTION

The novel coronavirus SARS-CoV-2 (COVID-19) infection that emerged in 2019 is the largest coronavirus infection in the world over the past 20 years. It spreads throughout population quickly and with devastating results. There is a global effort towards identifying the risk factors, clinical progression and best treatment practices for critically ill COVID-19 patients [1–4]. The WHO has listed COVID-19 as an international public health emergency and assigned a global risk assessment level as “Very High” [5].

The COVID-19 outbreak, initially originating in Wuhan, the Hubei province, has led to many deaths, but the clinical features of patients who died from COVID-19 pneumonia during the early stages of the outbreak are not well characterized. Since this information might have a profound value for COVID-19 treatment and epidemic control [6], this study analyzed the clinical characteristics of the first 17 reported cases of death due to COVID-19 pneumonia.

## RESULTS

### Demographic data

**Figure 1:**
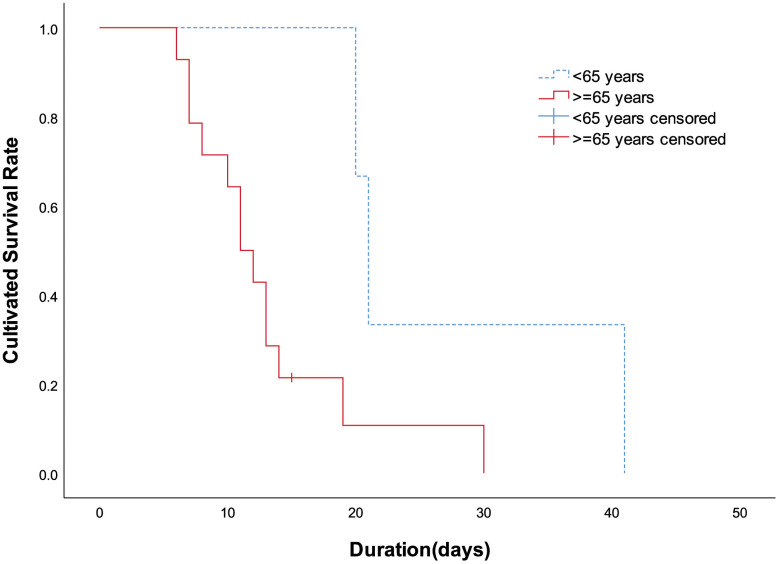
**Patients' cultivated survival rate between different age groups.** Compared with patients aged 65 years and over, the survival time less than 65 years was significantly longer (P=0.044 by Log-rank test).

The first known 17 death cases due to COVID-19 pneumonia were included in the study; they consisted of 13 males and 4 females. The age ranged from 48 to 89 years. Three cases (17.65%) were under 65 years old, seven cases (41.18%) were 65-80 years old, and seven cases (41.18%) were over 80 years old. The median age was 75 years. The survival time was significantly longer in patients younger than 65 years (P = 0.044, Figure 1). The average duration from hospital admission to death was 15.18 ± 9.05 days, with a range between 6 and 41 days; the median duration was 13 days. Within the first week of hospitalization, 1 (5.88%) patient died. Between days 7 and 14, 10 (58.82%) patients died. Six (35.29%) patients survived beyond 2 weeks after hospital admission.

### Underlying diseases

Out of the 17 patients, 7 (41.18%) did not report any history of an underlying disease. Among the 10 patients who had underlying diseases, 5 (50 %) exhibited more than two underlying diseases. Cardiovascular disease was the most common underlying disease, seen in 7 cases (70 %). The second most common underlying disease was cerebral infarction, seen in 3 cases (30 %). Two patients (20 %) had chronic obstructive pulmonary disease (COPD), and another 2 patients (20 %) had Parkinson's disease. The types and frequency of the underlying diseases are shown in Table 1.

**Table 1:** Clinical characteristic of COVID pneumonia deaths.

**Patients(n=17)**			**Patients(n=17)**
**Age (years)**	75 (66, 82)	**Complication**	
**Sex (male)**	13 (76.47%)	Ischemic stroke	1 (5.88%)
**Underlying disease**		Acute renal failure	1 (5.88%)
COPD	2 (11.76%)	Hepatic function damage	1 (5.88%)
Hypertension	7 (41.18%)	**Chest CT Reports**	
Diabetes	5 (29.41%)	Viral pneumonia	5 (29.41%)
Ischemic stroke	3 (17.65%)	Pulmonary interstitial infection	1 (5.88%)
CHD	2 (11.76%)	Ground glass shadow	2 (11.76%)
Coronary stenting	2 (11.76%)	Patch shadow	1 (5.88%)
Arrhythmia	1 (5.88%)	Pleural effusion	2 (11.76%)
Hyperuricemia	1 (5.88%)	Pleural adhesions	1 (5.88%)
Hyperlipidaemia	1 (5.88%)	Pulmonary fibrosis	1 (5.88%)
Parkinson disease	2 (11.76%)	Pulmonary nodule	1 (5.88%)
Chronic renal insufficiency	1 (5.88%)	Diffuse organic change	1 (5.88%)
Liver cirrhosis	1 (5.88%)	Bronchiectasia	1 (5.88%)
Gastrointestinal hemorrhage	1 (5.88%)	White lung change	1 (5.88%)
Myxoma	1 (5.88%)	**Treatment**	
Ascending aorta replacement	1 (5.88%)	Antiviral agent	3 (17.65%)
Abdominal aortic stent implantation	1 (5.88%)	Anti-infection	11 (64.71%)
Cholecystectomy	1 (5.88%)	Noninvasive ventilation	4 (23.53%)
Hip replacement	1 (5.88%)	CPR	1 (5.88%)
Colon cancer surgery	1 (5.88%)	Mechanical ventilation	9 (52.94%)
**Initial symptoms**		CRRT	1 (5.88%)
Cough	10 (58.82%)	Hepatic protector	1 (5.88%)
Expectoration	2 (11.76%)	Analgesia	1 (5.88%)
Dyspnea	7 (41.18%)	Sedative agents	2 (11.76%)
Nasal obstruction	1 (5.88%)	Vasoactive agents	4 (23.53%)
Fever	11 (64.71%)	Acidosis remedy	1 (5.88%)
Chill	1 (5.88%)	ECMO	1 (5.88%)
Fatigue	7 (41.18%)	**Duration (days)**	13 (10, 19)
Body aches	1 (5.88%)	**Outcome**	
Inappetence	1 (5.88%)	ARDS	16 (94.12%)
Headache	2 (11.76%)	Septic shock	3 (17.65%)
Confusion	1 (5.88%)	MODS	2 (11.76%)
Uracratia	1 (5.88%)		

COPD, chronic obstructive pulmonary disease; CHD, coronary heart disease; CPR, cardiopulmonary resuscitation; CRRT, continuous renal replacement therapy; ECMO, extracorporeal membrane oxygenation; ARDS, acute respiratory distress syndrome; MODS, multiple organ dysfunction syndrome.

### Initial symptoms and complications

Fever, fatigue, chills, and body aches were the most common initial symptoms, seen in 14 cases (82.35%). The second most common were respiratory symptoms, including cough, expectoration, dyspnea, and nasal obstruction, seen in 12 cases (70.59%). Three cases (17.65%) exhibited neurological symptoms including headache, confusion, and urinary incontinence. Digestive disorders, such as a lack of appetite were the rarest symptoms, seen only in 1 case (5.88%). Among the patients who did not provide a specific history of underlying disease, 1 patient (5.88%) developed cerebral infarction, 1 patient (5.88%) developed liver failure, and 1 patient (5.88%) developed kidney failure during the treatment. The initial symptoms and their frequency are shown in Table 1.

### Chest CT changes

Six out of 17 patients (35.29%) did not have chest CT results. Seven cases (63.64%) among the 11 patients who exhibited changed chest CT images had one change, such as viral pneumonia. Two (18.18%) patients had either 2 or 3 imaging changes. The chest CT image changes and their frequency are shown in Table 1.

### Treatments and outcomes

Three out of the 17 patients (17.65%) did not have any treatments beyond oxygen inhalation, expelling phlegm, and antipyretic drugs. From the 14 patients in the treatment group, 6 (42.86%) patients had 1 or 2 treatments, and 8 (57.14%) patients had 3 to 5 treatments (Table 1).

Among the 17 cases, 4 (23.53%) patients presented with 2 different fatal pathological and physiological states. One (5.88%) patient died of septic shock. The remaining 16 (94.12%) patients died of ARDS or a combination of ARDS and another condition (Table 1).

### Correlation analysis between clinical data

Age positively correlated with hypertension (r = 0.574, P < 0.05), and negatively correlated with the disease course (r = -0.539, P < 0.05), cough (r= -0.501, P < 0.05), anti-infection (r = -0.541, P < 0.05), vasoactive agents (r = -0.567, P < 0.05), and treatment quantities used (r = -0.774, P < 0.01). The disease course positively correlated with cough (r = 0.709, P < 0.01), mechanical ventilation (r = 0.566, P < 0.05), sedative treatment (r = 0.560, P < 0.05), treatment quantity (r = 0.511, P < 0.05), and sepsis shock (r = 0.505, P < 0.05). History of COPD positively correlated with chest CT diagnosis of pulmonary fibrosis (r = 0.685, P < 0.01) and nodules (r = 0.685, P < 0.01).

Cough positively correlated with fever (r = 0.633, P < 0.01), quantity of the initial symptoms (r = 0.754, P < 0.01), anti-infection (r = 0.633, P < 0.01), and quantity of treatments (r = 0.550, P < 0.05). CT diagnosis of viral pneumonia positively correlated with multiple organ dysfunction syndrome (MODS) (r = 0.566, P < 0.05). Both ground-glass opacity and pleural effusion negatively correlated with ARDS incidence (both r = -0.685, P < 0.01). Correlation between the clinical data is shown in Table 2.

**Table 2:** Spearman correlation analysis between partial clinical data.

	**Age**	**Duration**	**COPD**	**Ischemic stroke**	**Chronic renal insufficiency**	**Quantity of underlying disease**	**Cough**	**Expectoration**	**Fever**	**Chill**	**Headache**	**Confusion**	**Uracratia**	**Quantity of initial symptoms**	**Acute renal failure**
**Viral pneumonia**	-0.053	-0.462	0.165	-0.299	0.387	-0.055	-0.247	-0.236	0.207	-0.161	0.165	-0.161	-0.161	-0.272	0.387
**Pulmonary interstitial infection**	0.000	0.102	-0.091	-0.116	-0.063	0.080	0.209	0.685^*^	0.185	-0.063	0.685^*^	-0.063	-0.063	0.421	-0.063
**Ground glass shadow**	0.149	0.075	-0.133	-0.169	-0.091	-0.019	-0.065	-0.133	-0.112	-0.091	-0.133	-0.091	-0.091	-0.154	-0.091
**Patch shadow**	0.383	0.026	-0.091	0.540^#^	-0.063	0.080	-0.299	-0.091	-0.339	-0.063	-0.091	1.000^*^	1.000^*^	-0.105	-0.063
**Pleural effusion**	0.205	0.131	-0.133	0.310	-0.091	-0.136	-0.065	-0.133	-0.112	-0.091	-0.133	.685^*^	.685^*^	0.000	-0.091
**Pleural adhesions**	0.383	0.026	-0.091	0.540^#^	-0.063	0.080	-0.299	-0.091	-0.339	-0.063	-0.091	1.000^*^	1.000^*^	-0.105	-0.063
**Pulmonary fibrosis**	-0.179	-0.205	.685^*^	-0.116	1.000^*^	0.373	0.209	-0.091	0.185	-0.063	0.685^*^	-0.063	-0.063	0.316	-0.063
**Pulmonary nodule**	-0.179	-0.205	0.685^*^	-0.116	1.000^*^	0.373	0.209	-0.091	0.185	-0.063	0.685^*^	-0.063	-0.063	0.316	-0.063
**Diffuse organic change**	-0.409	0.409	-0.091	0.540^#^	-0.063	0.080	0.209	0.685^*^	0.185	-0.063	-0.091	-0.063	-0.063	0.316	-0.063
**Bronchiectasia**	-0.409	0.409	-0.091	0.540^#^	-0.063	0.080	0.209	0.685^*^	0.185	-0.063	-0.091	-0.063	-0.063	0.316	-0.063
**White lung change**	-0.179	0.358	-0.091	-0.116	-0.063	-0.266	0.209	-0.091	-0.339	-0.063	-0.091	-0.063	-0.063	-0.316	-0.063
**Antiviral agent**	0.000	-0.268	0.310	-0.214	0.540^#^	0.115	0.074	0.310	0.019	0.540^#^	.789^*^	-0.116	-0.116	0.390	-0.116
**Anti-infection**	-0.541^#^	0.340	0.270	0.019	0.185	-0.026	0.633^*^	0.270	0.485^#^	0.185	0.270	-0.339	-0.339	0.414	-0.339
**Noninvasive ventilation**	-0.425	0.099	0.228	0.107	0.451	-0.044	0.182	0.228	0.119	0.451	0.228	-0.139	-0.139	0.350	-0.139
**CPR**	-0.256	-0.128	-0.091	-0.116	-0.063	-0.266	-0.299	-0.091	-0.339	-0.063	-0.091	-0.063	-0.063	-0.105	-0.063
**Mechanical ventilation**	-0.386	0.566^#^	-0.387	0.127	-0.265	-0.138	0.408	0.344	0.290	-0.265	-0.022	0.236	0.236	0.595^#^	-0.265
**CRRT**	-0.102	0.153	-0.091	-0.116	-0.063	-0.266	0.209	-0.091	0.185	-0.063	-0.091	-0.063	-0.063	0.105	-0.063
**Hepatic protector**	-0.102	0.153	-0.091	-0.116	-0.063	-0.266	0.209	-0.091	0.185	-0.063	-0.091	-0.063	-0.063	0.105	-0.063
**Analgesia**	-0.409	0.409	-0.091	.540^#^	-0.063	0.080	0.209	0.685^*^	0.185	-0.063	-0.091	-0.063	-0.063	0.316	-0.063
**Sedative agents**	-0.429	0.560^#^	-0.133	0.310	-0.091	-0.136	0.306	0.433	-0.112	-0.091	-0.133	-0.091	-0.091	0.000	-0.091
**Vasoactive agents**	-0.567^#^	0.326	-0.203	-0.257	-0.139	-0.399	0.182	-0.203	0.119	-0.139	-0.203	-0.139	-0.139	0.117	-0.139
**Acidosis remedy**	-0.307	0.256	-0.091	-0.116	-0.063	0.080	0.209	-0.091	0.185	-0.063	-0.091	-0.063	-0.063	0.105	-0.063
**ECMO**	-0.307	0.256	-0.091	-0.116	-0.063	0.080	0.209	-0.091	0.185	-0.063	-0.091	-0.063	-0.063	0.105	-0.063
**Quantity of treatment**	-0.774^*^	0.511^#^	-0.019	0.000	0.157	-0.220	0.550^#^	0.382	0.309	0.157	0.229	-0.183	-0.183	0.606^*^	-0.366
**ARDS**	0.102	-0.153	0.091	0.116	0.063	0.266	-0.209	0.091	-0.185	0.063	0.091	0.063	0.063	-0.105	0.063
**Septic shock**	-0.394	0.505^#^	-0.169	-0.214	-0.116	-0.493^#^	0.387	-0.169	0.019	-0.116	-0.169	-0.116	-0.116	-0.065	-0.116
**MODS**	-0.056	-0.392	0.433	-0.169	0.685^*^	0.078	-0.065	-0.133	0.270	-0.091	0.433	-0.091	-0.091	0.000	0.685^*^

COPD, chronic obstructive pulmonary disease; CHD, coronary heart disease; CPR, cardiopulmonary resuscitation; CRRT, continuous renal replacement therapy; ECMO, extracorporeal membrane oxygenation; ARDS, acute respiratory distress syndrome;MODS, multiple organ dysfunction syndrome.

## DISCUSSION

This study analyzed the first 17 reported deaths from COVID-19 infection. Our data showed that increased age and male gender were the main predictors of death, consistent with previous findings [1, 7–12]. Hypertension positively correlated with advanced age among patients who died from COVID-19. Age negatively correlated with duration of symptoms, cough, anti-inflammation therapy, vasoactive agents, or increased number of treatments. Median survival time of the 17 patients was only 13 days. The disease course was related to cough, sedation treatment, treatment quantity, and septic shock. Patients with cough as the first symptom were more likely to have a longer duration of disease, take sedation treatment, and have a high risk of death from septic shock at the late stage of the disease.

This cohort of 17 patients had the comorbidity rate of 41.18%, with cardiovascular diseases followed by COPD. This observation is consistent with previously observed epidemiological characteristics of the disease prevalence in China's elderly population [13]. The presence of an underlying disease increases the risk of poor coronavirus prognosis [7–9, 14]. For example, COPD with pulmonary nodules and pulmonary fibrosis exacerbates the prognosis for severe pneumonia from COVID-19 [14, 15]. Similarly, patients with neurological deficits caused by ischemic stroke are more prone to confusion and incontinence [14]. Furthermore, a history of chronic renal insufficiency, ascending aortic artery replacement, abdominal aortic stent implantation, or cholecystectomy positively correlated with MODS and a poor prognosis in COVID-19 severe pneumonia [16].

The most common systemic symptoms included fever and fatigue, followed by respiratory symptoms, such as cough and dyspnea, and nervous system symptoms. These trends suggest that if elderly coronavirus patients develop systemic or respiratory symptoms, there is an urgent need for effective early treatments to improve their prognosis [7, 8, 10, 17–19]. Fever and cough are common symptoms of severe pneumonia; they correlated with the extent of initial COVID-19 symptoms and anti-infection treatment. Therefore, COVID-19 patients with fever and cough are more likely to exhibit multiple symptoms and take anti-infection treatment. There is a strong correlation between phlegm production, lung interstitial infection, diffuse organic change, bronchial dilation, and chest imaging changes in patients with severe pneumonia [20–22]. Nasal obstruction, lack of appetite, and headache positively correlated with CT pulmonary interstitial infection and antiviral treatment, suggesting that COVID-19 infection might have caused the observed lung interstitial changes and prompted the antiviral treatment.

Chronic renal insufficiency increases the risk of acute renal failure, and they both increase the risk of MODS. Therefore, in severe pneumonia, both acute and chronic renal insufficiency can be used as a predictor of MODS complications. Furthermore, the extent of initial COVID-19 symptoms positively correlated with the need for mechanical ventilation and other treatments. Majority of CT scans among this cohort were indicative of viral pneumonia. However, there were many differences in the CT results, such as ground- glass opacity, patch shadows, and white lung changes. Our findings are similar to other reports of CT imaging changes among COVID-19 patients [23, 24]. We hypothesize that the positive correlation between CT changes with viral pneumonia, pulmonary nodule, pulmonary fibrosis, and MODS may result from acute or chronic renal insufficiency. We also hypothesize that the negative correlation found between ground-glass opacity or pleural effusion and ARDS may be due to viral pneumonia. We rarely observed pleural effusion; this is consistent with the National Health Commission’s coronavirus diagnostic guidelines [25].

Most COVID-19 patients have respiratory distress that requires assistance in breathing. Patients in critical condition require high frequency mechanical ventilation and non-invasive ventilation. Early breathing support may improve prognosis of COVID-19 patients [8]. ARDS was the leading cause of death in our cohort; this is consistent with the pathological and physiological characteristics of patients with severe pneumonia [7–9, 17]. As the severity of pneumonia increases, so does the risk of ARDS. For example, the incidence rate of ARDS in general population is between 19.60% and 29.00%, and in intensive care units between 61.10 % and 85.00 %; however, it was 94.12% in this study. Thus, an early intervention to reduce the incidence of ARDS may improve the prognosis of COVID-19 pneumonia patients. In addition, some COVID-19 patients had septic shock or MODS, which aggravated the patient's condition and shortened the time from onset to death; this is consistent with previous findings [7].

This study is not without limitations, which include its rather limited scope. For example, we did not have access to blood tests or the specific therapeutic drug history that might have affected the diagnosis and treatment. Secondly, the data were limited in size, and increased sample size would increase the statistical power and ability for generalization. However, the focus of this study was to characterize the first reported cases of deaths due to COVID-19 in China. Since SARS-CoV-2/COVID-19 is a novel virus, it will require extensive studies to understand the mechanisms of its action [2, 4]. Additionally, the study was limited to early deaths with no control group established. Therefore, the disease characteristics compared with patients of mild symptoms could not be established and the effectiveness of disease treatment program could not be tested. Finally, even though China is no longer the most affected country in the world, it was the first country to identify the pathogen and control the epidemic, and thus the characteristics of the early death cases and countermeasures are important for the control of the epidemic in other countries.

## MATERIALS AND METHODS

Clinical data of 17 cases of early death in COVID-19 pneumonia patients in Wuhan were retrieved from the National Health Commission of China on January 23, 2020 [6]. Patient’s occupation, contact history and incubation period were not provided in the data. Data collection, analysis, and dissemination were performed by the National Health Commission; thus, the study is exempt from the institutional ethics review.

### Diagnostic criteria

The novel coronavirus infection treatment program issued by the National Health Commission of China on January 16, 2020 [25] was used to establish a diagnosis of COVID-19 at the beginning of the outbreak. The diagnostic criteria included: (1) History of travel in Wuhan including direct or indirect contact with the Wuhan markets, such as the farmer’s market, in the two weeks before the onset of symptoms. (2) Clinical symptoms of fever and chest imaging consistent with pneumonia. For example, in the early stages of the disease we would see multiple small plaques and interstitial changes, especially in the extraneous lung. As the disease progressed, multiple ground glass shadows or infiltration shadows appeared in both lung lobes. Consolidation occurred in severe cases, but was less likely with pleural effusion. (3) Results of whole genome sequencing of a respiratory specimen, such as sputum or a pharyngeal swab, that revealed the presence of a virus that shared homology with the novel coronavirus.

Data on age, sex, clinical history, first symptoms, image examination results, treatment programs, procedures, and causes of death were collected. A retrospective analysis was carried out on those data.

### Statistical methods

We used Spearman correlation coefficient to calculate correlative relationships. Statistical significance was set as P < 0.05. We used the Kaplan-Meier method to analyze survival curves. A log rank test was used to determine differences between survival curves. Data relating to how many times a characteristic appears in the dataset is presented as number (percentage of the whole). Quartile data is presented as median (upper quartile, lower quartile).

### Ethics approval and consent to participate

Data collection, data analysis, and information dissemination as the basis for the epidemic control were completed by the National Health Commission; thus, they were exempt from the institutional ethics review.

### Availability of data and materials

The authors have full rights to access and share the data upon appropriate usage.
